# Occupational injuries and contributing factors among industry park construction workers in Northwest Ethiopia

**DOI:** 10.3389/fpubh.2022.1060755

**Published:** 2023-01-11

**Authors:** Tewodros Yosef, Enawgaw Sineshaw, Nigusie Shifera

**Affiliations:** ^1^School of Public Health, College of Medicine and Health Sciences, Mizan-Tepi University, Mizan Teferi, Ethiopia; ^2^Department of Public Health, College of Health Sciences, Debre Markos University, Debre Markos, Ethiopia

**Keywords:** occupational injuries, construction workers, Bure, Amhara region, Ethiopia

## Abstract

**Background:**

Construction business is currently the second greatest source of injuries in Ethiopia after automotive accidents, with a risk of fatality that is five times higher than that of other industrial sectors. To establish measures for injury prevention, it is crucial to assess the severity of occupational injuries and identify the variables that contribute to them. Therefore, this study aimed to assess the magnitude and factors associated with occupational injuries among Bure Industrial Park construction workers, Northwest Ethiopia.

**Methods:**

An institutional-based cross-sectional study was conducted among 372 construction workers at Bure Industrial Park. The study participants were selected using a simple random sampling method. The data were collected using interviewer-administered structured questionnaire and work environment observation using structured checklist. In the descriptive statistic, frequencies, proportion, and mean were calculated and the results of the analysis were presented in text and tables. The bi-variable and multivariable logistic regression analyses were carried out to identify independent factors having associations with the occurrence of occupational injury.

**Results:**

The overall prevalence of occupational injuries among Bure industrial park construction workers was 39.4%, 95%C.I (34.4%-44.4%). Factors such as sex (being male) [AOR = 1.74, 95%CI (1.02–2.97)], being married [AOR = 2.79, 95%CI (1.50–5.17)], no use of personal protective equipment [AOR = 1.67, 95%CI (1.12–2.85)], no training on occupational safety [AOR = 1.45, 95%CI (1.06–2.98)], and not satisfied with the job [AOR = 5.97, 95%CI (3.48–10.2)] were the factors associated with occupational injuries.

**Conclusion and recommendation:**

The finding shows the public health importance of occupational injury among construction workers in the study area. Numerous factors have been linked to workplace injuries, including sex, marital status, the usage of personal protection equipment, training in occupational safety, and job satisfaction. As a result, in order to lower the rate of occupational injury, employers should prioritize offering safety training, encouraging the use of personal protective equipment while working, conducting routine workplace inspections, and ensuring that their staff members are happy at work by providing comfortable workspaces.

## Introduction

Any accidental physical hurt or harm to body tissue resulting from work exposure is referred to as an occupational injury (1). The construction sector is home to about a quarter of all work-related fatalities, in addition to a large number of other injuries (2). Safety in the workplace is described as preventing incidents that could cause bodily harm to people (3, 4). Safety on construction sites lowers the danger of work-related accidents and injuries as well as harm to the general public (2).

Worldwide, hundreds of millions of individuals are working in unsafe circumstances (5). In both industrialized and developing countries, work injuries have been identified as one of the most important factors that contribute to poor health and life-threatening illnesses (6). Globally, there are approximately 270 million occupational injuries and two million fatalities per year in all productive sectors (6, 7). Occupational illnesses or accidents claim the lives of 6,300 people per day (8). Annually, 55,000 fatal injuries are caused by the construction sector (9). According to estimates, occupational accidents cost the global economy 4% of its GDP (USD 1.25 trillion) (10, 11). The annual projected direct and indirect costs of fatal and nonfatal construction injury are above $10 billion USD (12).

Construction industry is one of the riskiest and most accident-prone industries (6, 13). People working on construction sites are more likely to suffer traumatic injuries, illnesses, and fatalities than people in other occupations in both developed and developing countries (12, 14–16). Compared to manufacturing industry, peoples working in construction have 2.5 and 5 times higher risk of serious injury and death respectively (17).

Construction workers in poor countries experience occupational health and safety risks that are between 10 and 20 times more severe than those in industrialized countries (18). Construction workers have greater occupational health and safety injuries in developing countries than in developed ones; this is more common in Sub-Saharan Africa. This could be because there are less laws and regulations governing workplace health and safety (19).

The magnitude of work injuries among construction employees in Egypt was reported to be 46.2% (20), 74% in Kenya (21), and the magnitude range from 38.3 and 84.7% in Ethiopia (10, 22). Although unsafe work environments are frequently the cause of workplace injuries, other factors are also mentioned as contributing to occupational injuries, including sex, age, workload, lack of safety training, job stress, the absence of safety signs, sleep issues, alcohol consumption, cigarette smoking, chewing khat, and poor exercise habits (10, 13, 21–23).

Construction business is currently the second greatest source of injuries in Ethiopia after automotive accidents, with a risk of fatality that is five times higher than that of other industrial sectors (7). Although evidence-based work health and safety services are essential, studies showing the prevalence and factors of occupational injuries in the Construction Park are scarce in Ethiopia, particularly no study conducted in the study area so far. Accident prevention starts with having a firm awareness of the contributing factors because accident causalities in the construction sector are complicated and multidimensional (24). To establish measures for injury prevention, it is crucial to assess the severity of occupational injuries and identify the variables that contribute to them. Therefore, this study aimed to assess the magnitude and factors associated with occupational injuries among Bure Industrial Park construction workers, Northwest Ethiopia.

## Methods

### Study design, area, and period

An institutional-based cross-sectional study was in Bure industrial park construction workers from January to February 2022. Bure town administration Industry Park is found in the Amhara region, 411 km Northwest of Addis Ababa, the capital city of Ethiopia. The park has created job opportunities for thousands of people in the area. The number of workers fluctuates from time to time from a minimum of 1,500 to a maximum of 2,000. These construction projects cover range of activities such as site clearance, the demolition or dismantling of building structures or plants and equipment, excavations, reinforcement-bar works, concrete works, HCB (Hollow Concrete block) other material fabrication, decoration, cleaning, installation, and the removal and maintenance of services (electricity, water, and telecommunications). It also includes the use of woodworking, painting, and decorating and the use of heavy machinery for site landscaping.

### Populations

All Bure industry park workers were the source population. The study populations were all randomly selected Bure industry park, construction workers. All Bure industrial park construction workers were eligible and included regardless of their job categories whether they working as daily labor, plasterer, carpenter, mason, welder/electrician, painter, driver/operator, and office/site engineers under construction Enterprise. Construction workers who were unable to respond due to illness and workers with hearing or speaking difficulties were excluded.

### Sample size determination and sampling technique

The sample size was calculated using a single population proportion formula by assuming 32.6% the prevalence rate of occupational injuries among construction workers in Dessie town (11), with a 95% confidence level, 5% desired precision, and adding 10% for non-response rate, the total calculated sample size was 372. First, a list of building construction works with their respective job category was obtained from Bure Industrial park administration. Then, a simple random sampling technique was employed to select the study participants. If the selected participant is not available at the time of data collection, the next participant was considered.

### Study variables

The presence of occupational injuries among construction workers was the outcome variable. The independent variables were socio-demographic and economic factors (age, residence, marital status, economic status, educational level, medical condition, pattern of employment, salary), occupational factors (working section/job category, total work hours/day, availability of safe tools, availability safe machinery, occupational safety training, availability PPE (personal protective equipment's) and behavioral factors (use of PPE **(**personal protective equipment's), job satisfaction, sleeping disturbance problem, usage of substances).

### Operational definitions

Occupational injuries are any physical injuries sustained by a worker in connection with the performance of his or her work (25). Personal Protective Equipment (PPE) was defined as specialized clothing or equipment worn by employees for protection against health and safety hazards. Workers were classified as those who used PPE when they responded to use PPE that was necessary to be worn during a particular activity (10). Substance use was defined as a person who used at least one of the following substances such as cigarette, khat and alcohol in the past 30 days (26).

### Data collection tools, procedures, and quality assurance

The data were collected using a pretested structured questionnaire, which was developed after reviewing relevant literature (11, 14). After preparing the English version it was translated first into Amharic and then back to English to keep its consistency. The local language Amharic was used to collect the data. A face-to-face interview was used to collect the data. The questionnaire was composed of the following variables; socio-demographic and economic factors (age, residence, marital status, economic status, educational level, medical condition, pattern of employment, and salary), occupational factors (working section/job category, total work hours/day, availability of safe tools, availability safe machinery, occupational safety training, availability PPE (personal protective equipment's), behavioral factors [use of PPE **(**personal protective equipment's), job satisfaction, sleeping disturbance problem, usage of substances], and occupational injury-related variables (the occurrence of injuries, and their types, time of injury happen and causes of injury). The outcome variable was occupational injury. It was measured by asking respondents a question stated as, “Have you encountered any injuries in the past 12 months?” Responses that were “yes” were coded as “1” while responses that were “no” were categorized as “0”. In addition to participant self-reports, we confirm the existence and type of injury by looking at the respondent's damaged body part. The health center record also used to confirm such injuries which are documented when an individual's sustain injury and visit for treatment. The availability and use of personal protective equipment (PPE) as well as different workplace hazards (such as whether respondents worked with machines or not and whether they worked in an environment that made them vulnerable to injury) were also determined using a work environment observation checklist. The questionnaire's face validity was examined by professionals in occupational health. In terms of instrument reliability, a test of reliability was conducted on the questionnaire status, and a satisfactory reliability status was obtained (in this study, a Cronbach's alpha of 0.79 was obtained). A pretest of the tool was conducted on 5% of the sample size (not actually part of the study, but had similar characteristics) among construction workers in Finote Selam town before the actual data collection commenced and necessary correction was done. The data collection was done by three BSc nurses, who had previous experience in data collection. The overall data collection process was supervised by two BSc public health officers. Two days of training was given for data collectors along with their supervisors about the questionnaire and data collection procedures.

### Data processing and analysis

The data were coded and entered into Epi Data version 3.1 and then exported to SPSS version 20 for statistical analysis. In the descriptive statistic, frequencies, proportion, and mean were calculated and the results of the analysis were presented in text and tables. Binary logistic regression analysis was carried out to assess the association of different independent variables with the dependent variable. Independent variables having *P* < 0.25 on the binary logistic regression analysis were considered as candidates for the final multivariable logistic regression analysis. The level of significance was declared at a *p* < 0.05.

### Ethical approval and consent to participate

Ethical approval was obtained from Mizan-Tepi University Ethical Review Committee. Confidentiality and privacy were maintained; only the ID number was used during data collection, analysis, and reporting in which the information obtained from the respondents will not be shared with anyone other than the data collectors and principal investigator. The data collectors provide health education related to occupational injuries to study participants a long side of the data collection process. Taking the current COVID-19 pandemic into account, preventive measures such as the use of personal protective materials and physical distancing were applied during the data collection. First aid kit and other necessary materials were prepared in advance to provide first aid service for the participants if occupation accidents occur at the time of data collection. Written informed consent was obtained from participants who participated in the study.

## Results

### Socio-demographic characteristics

Of the 377 total sample size, 368 study participants have completed the interview, giving a response rate of 97.6%. The mean age of the participants was 27.2 (±8.4 SD) ranging from 16 to 47 years old. Almost all of the respondents, 348 (94.5%) and 348 (94.8) were orthodox religion followers and Amhara by ethnicity respectively (Table 1). The mean monthly salary of the participants was 3,444 (±1,449 SD) ranging from 1,800 to 6,000 Ethiopian birr.

**Table 1:** Socio-demographic characteristics of the Bure industry park construction workersin Northwest Ethiopia.

**Variables**	**Categories**	**Frequency**	**Percent**
Sex	Male	179	48.6
Age	Female	189	51.4
	≤ 25 years	74	20.1
	26–35 years	184	50
	36–45 years	92	25
	≥ 46 years	18	4.9
Religion	Orthodox	348	94.5
	Protestant	8	2.2
	Muslim	12	3.3
Ethnicity	Amhara	349	94.8
	Oromo	11	3
	Others^*^	8	2.2
Marital status	Out of marriage	199	54.1
	Married	169	45.9
Educational level	Illiterate	8	2.2
	Able to read and write	4	1.1
	Primary school (1–8)	46	12.5
	Secondary (9–12)	74	20.1
	College and above	236	64.1
Previous residence	Urban	362	98.4
	Rural	6	1.6
Monthly salary (ETB)	< 3,444	196	53.3
	≥3,444	172	46.7

* Others: South and Tigre; ETB, Ethiopian Birr.

### Description of the pattern of occupational injuries

**Figure 1:**
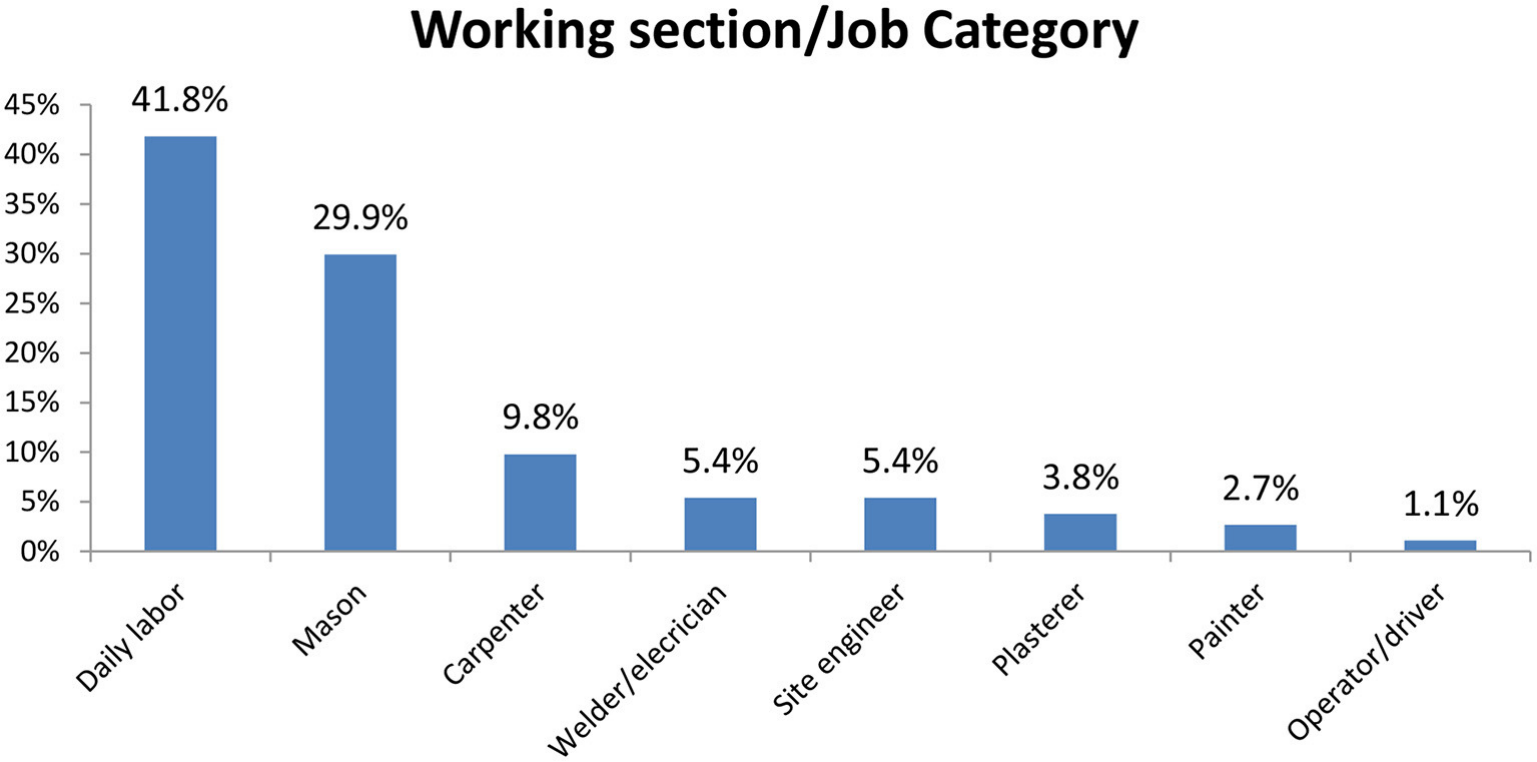
The working section/job category of the Bure industry park construction workers in Northwest Ethiopia.

Of the 368 study participants, 41.8% were daily laborers, followed by 29.9% masons and 9.8 carpenters (Figure 1). The commonest type of injury mentioned was laceration (42%) followed by 23.5%. One hundred seventy-six (47.8%) used personal protective equipment (Table 2). The overall prevalence of occupational injuries among workers of Bure industrial park was 39.4%, 95% C.I (34.4–44.4%).

**Table 2:** Description of the occupational injury-related characteristics among Bure industry park construction workers in Northwest Ethiopia.

**Variables**	**Categories**	**Frequency**	**Percent**
Have you ever transferred from one working section to other? (*n* = 368)	Yes	36	9.8
	No	332	90.2
Reason for your transfer (*n* = 36)	Health problem	4	11.1
	Workload	6	16.1
	For better salary	26	72.2
Total working hours per week (*n* = 368)	< 48 h	66	17.9
	48 h	237	64.4
	≥48 h	65	17.7
Have you encountered any injuries in the past 12 months? (*n* = 368)	Yes	145	39.4
	No	223	60.6
Number of times in the past 12 months (*n* = 145)	Once	58	40
	Two or more	87	60
Time of injury happen (*n* = 145)	Morning	50	34.5
	Afternoon	95	65.5
Kinds of injury (*n* = 145)	Laceration	62	42.8
	Stab	34	23.5
	Fracture	29	20
	Joint dislocation	20	13.7
Causes of injury (*n* = 145)	Personal failure	41	28.3
	Occupational failure	104	71.7
Personal protective equipmentuse (*n* = 368)	Yes	176	47.8
	No	192	52.2
Type of treatment after injury (*n* = 145)	First aid	63	43.5
	General treatment	82	56.5

### Common work-related determinants of injuries and behavioral profiles

Fifty-four (14.7%) of the participants had occupational safety training at work. More than three-fourths (80.4%) of the participants had regular workplace supervision. Slightly above half (47.8%) and 36 (9.8%) of the participants were alcohol drinkers and had sleep disturbance, respectively (Table 3).

**Table 3:** Common occupational and behavioral factors of injuries among Bure industry park construction workers in Northwest Ethiopia.

**Variables**	**Categories**	**Frequency**	**Percent**
Training on occupational safety	Yes	54	14.7
	No	314	85.3
Regular workplace supervision	Present	296	80.4
	Absent	72	19.6
Work done by using machinery	Yes	298	91
	No	70	19
Machinery design in a way that does not cause accident and injury (n=298)	Yes	236	79.2
	No	62	20.8
Alcohol drinking	Yes	176	47.8
	No	192	52.2
Cigarette smoking	Yes	66	17.9
	No	302	82.1
Khat chewing	Yes	40	10.9
	No	328	89.1
Do you hand a sleeping disturbance problem?	Yes	32	9.8
	No	336	90.2

### Observational finding

After reviewing the records, 112 (30.4%) of the respondents visited the health center for treatment after sustained injury. Of 112 participants visited health center, the majority, 35 (31.3%) for laceration followed by 34 (30.4%), 29 (25.9%) and 14 (12.5%) were for stab, fracture and dislocation injuries respectively. Despite not being full, all participants had at least one PPE. One hundred seventy-six (47.8%) of the participants used personal protective equipment at work (Table 2). Two hundred ninety-eight (91%) of the participants were work with machineries. Of those who work with machineries, 62 (20.8%) were work under risky machineries (Table 3).

### Factors associated with occupational injuries

After A multi variable logistic regression four variables namely; sex (being male) [AOR = 1.74, 95%CI (1.02–2.97)] being married [AOR = 2.79, 95%CI (1.50–5.17)], no use of personal protective equipment [AOR = 1.67, 95%CI (1.12–2.85)], no training on occupational safety [AOR = 1.45, 95%CI (1.06–2.98)], and not satisfied with the job [AOR = 5.97, 95%CI (3.48–10.2)] were the factors associated with occupational injury (Table 4).

**Table 4:** Factors associated with occupational injuries among Bure industry park construction workers in Northwest Ethiopia (*n* = 368).

**Variables**	**Categories**	**Occupational injuries**		**COR (95% CI)**	**AOR (95% CI)**	***P*** **-value**
		**Yes**	**No**			
Sex	Female	59	130	1	1	
	Male	86	93	2.04(1.48–3.47)^**^	1.74(1.02–2.97)	**0.042**
Marital status	Out of marriage	67	132	1	1	
	Married	78	91	1.69(1.11–2.58)^*^	2.79(1.50–5.17)	**0.001**
Income	< 3,444	62	134	1	1	
	≥3,444	83	89	2.02(1.32–3.08)^**^	0.83(0.43–1.58)	0.567
Personal protective equipment use	Yes	55	121	1	1	
	No	90	102	1.94(1.27–2.97)^**^	1.67 (1.12–2.85)	**0.032**
Training on occupational safety	Yes	29	25	1.98(1.11–3.54)^**^	1.45(1.06–2.98)	**0.017**
	No	116	198	1	1	
Job satisfaction	Yes	91	51	1	1	
	No	54	172	5.68(3.59–8.99)^**^	5.97(3.48–10.2)	**< 0.001**

AOR, adjusted odds ratio; CI, confidence Interval; COR, crude odds ratio.

* p < 0.05,

** p < 0.01.

The bold values are used to show the level of significance. All bold values are significant variables.

## Discussion

Construction business is one of the most dangerous workplaces (15). It is currently the second greatest source of injuries in Ethiopia after automotive accidents, with a risk of fatality that is five times higher than that of other industrial sectors (7). Although evidence-based work health and safety services are essential, studies showing the prevalence and factors of occupational injuries in the Construction Park are scarce in Ethiopia, particularly no study conducted in the study area so far. Therefore, this study aimed to assess the magnitude and factors associated with occupational injuries among Bure Industrial Park construction workers in Northwest Ethiopia.

The prevalence of occupational injury in the last year among Bure industry park construction workers was 39.4%, 95% C.I (34.4–44.4%). The prevalence stated above was consistent with 39% in Gonder (15), 39.2% in Robe town (27), 38.3% in Addis Ababa (10) studies in Ethiopia, and 39.3% in Nigeria (15). The result of this study is lower than 84.7 and 67.7% in Addis Ababa, Ethiopia (20, 22), 79.8% in Iran (28), and 46.2% in Egypt (20). The result of this study is also higher than 15% in Gondar, Ethiopia (29), 32.4% in Uganda (30), and 34.8% in China (31). The possible discrepancy may be due to study setting differences, working conditions, level of accident prevention strategies, and socio-cultural and regulatory factors (15). Besides, the difference in the level of regular workplace supervision, PPEs utilization, and working hours per day, as well as week, may create a considerable variation across different studies. There was also a discrepancy between the self-reported and observation of the health center records. This could be due to the participants' avoidance of seeking care for laceration by considering it as a mild. Besides, the preference and attendance of traditional healers for joint dislocation also another thing for the variation observed. The incompleteness of documentation due to the above reasons, the discrepancy between self-report and record observation was observed.

Male construction workers were more vulnerable to occupational injury than female construction workers. In this study, the risk of occupational injury was 1.74 times higher among male workers as compared to female workers. This finding was consistent with a studies conducted in Ethiopia (31–33), Ghana (34), and China (35) which reported that male workers are more prone to occupational injury than female workers. The possible explanation for this report is due to the difference in tasks and males are high in risk-taking behavior (36).

The occurrences of occupational injury among married were 2.8 times higher as compared to their counterparts. The finding of this study is supported by another similar study conducted in Gonder, Ethiopia (15) and Iran (37). This may be due to married workers may more engage in works without taking adequate rest to cover a family expenses. Stress and fatigue can be higher among married workers than single ones because of higher responsibilities in life to secure family needs. It may be led to more unsafe acts resulting in an accident (32).

The odds of work-related injuries among workers who did not receive occupational safety training were 1.45 times more likely compared to those who received occupational safety training. This finding was supported by a study done in Bahirdar (1), Dessie (11), and a systematic analysis in Ethiopia (32), which reported that workers who attend safety training programs were less likely to experience work-related injuries. This might be due to training that provides knowledge about the presence of different safety hazards in construction and helps workers how to protect them. In addition, training may have an impact on changing the behaviors of workers to follow the safety precautions.

Personal protective equipment use was another factor significantly associated with the occurrence of occupational injuries. Accordingly, workers who did not engage in the work by wearing personal protective equipment were 1.67 times less likely to develop occupational injuries than those workers who did use PPE. This finding was supported by studies done in Bahrdar, Ethiopia (11), and Addis Ababa, Ethiopia (20), Uganda (30), which indicated that the use of PPE in a working environment reduces the occurrence of occupational injuries. This could be due to personal protective equipment protecting the worker against the hazards to which the worker may be exposed. Worker protection equipment (PPE) shields workers against a range of dangers, including chemical, physical, biological, electrical, mechanical, and radiological dangers (38).

Workers who had no job satisfaction were a 5.6 times higher risk of developing occupational injuries compared to their counterparts. This finding was supported by a study conducted in Addis Ababa, Ethiopia (20), and Nigeria (15). This might be because those workers who had no satisfaction with work did not comply with standard work procedures, and safety precautions including proper use of PPEs. Evidence suggests that there is a link between accidents and Job satisfaction (39). Job satisfaction can result in improved performance and a decrease in occupational accidents (40). This means that job dissatisfaction might lead to an increase unsafe acts and result in occupational accidents (41).

## Limitations of the study

One-year prevalence may be underestimated or overestimated due to recall and social desirability bias although much effort was taken to minimize it. Furthermore, the study's cross-sectional design makes it difficult to demonstrate cause-and-effect linkages between the dependent and independent variables. Therefore, future studies should be consider with better study designs (cohort study) to minimize recall bias and to determine the cause-and-effect relationship between the dependent and independent variables.

## Conclusion and recommendation

The finding shows the public health importance of occupational injury among construction workers in the study area. Numerous factors have been linked to workplace injuries, including sex, marital status, the usage of personal protection equipment, training in occupational safety, and job satisfaction. As a result, in order to lower the rate of occupational injury, employers should prioritize offering safety training, encouraging the use of personal protective equipment while working, conducting routine workplace inspections, and ensuring that their staff members are happy at work by providing comfortable workspaces.

## Data availability statement

The raw data supporting the conclusions of this article will be made available by the authors, without undue reservation.

## Ethics statement

The studies involving human participants were reviewed and approved by Mizan-Tepi University Ethical Review Committee. The patients/participants provided their written informed consent to participate in this study.

## Author contributions

All authors made a significant contribution to the work reported, whether that is in the conception, study design, execution, acquisition of data, analysis, and interpretation, or in all these areas, took part in drafting, revising, or critically reviewing the article, gave final approval of the version to be published, have agreed on the journal to which the article has been submitted, and agreed to be accountable for all aspects of the work.
